# Resolved HBV behavior during the treatment of chronic HCV infection with direct-acting antivirals 

**Published:** 2019

**Authors:** Salem Y. Mohamed, Baasim A. Gaballah, Hany Mohamed Elsadek, Mohamed Hassan Emara, Emad F. Hamed

**Affiliations:** 1 *Gastroenterology and Hepatology Unit, Internal Medicine Department, Faculty of Medicine, Zagazig University, Zagazig, Egypt*; 2 *Endocrine unit, Internal Medicine Department, Faculty of Medicine, Zagazig University, Zagazig, Egypt*; 3 *Department of Gastroenterology, Hepatology and Infectious Diseases, Kafr Elsheik University, Kafr Elsheik, Egypt *

**Keywords:** HCV infection, Resolved HBV, HBV flare, Direct-acting antivirals

## Abstract

**Aim:**

This paper aimed to assess and follow up the course of resolved HBV (hepatitis B virus) during and after treatment with direct-acting antiviral drugs (DAAs).

**Background:**

Co-infection with hepatitis B and hepatitis C is increasingly recognized in patients with chronic hepatitis. Resolved HBV in patients with chronic HCV (hepatitis C virus) infection has been investigated during interferon therapy, and the investigators suggest a possible correlation with a lower response to anti-viral treatment, higher grades of liver histological changes, and development of hepatocellular carcinoma.

**Methods:**

Three hundred and thirteen patients were included in our observational and prospective study; two hundred and fifty-three patients had chronic hepatitis C (CHC) (group I), and sixty patients had both CHC and resolved HBV-infection (group II). They all were eligible for treatment with DAAs therapy for chronic HCV in our hepatology unit, Internal Medicine Department, Zagazig University Hospitals from December 2017 to September 2018. They were subjected to thorough history taking, full clinical examination, routine laboratory investigations, HCV antibody, HCV RNA, HBV surface antigen (HBsAg), HBV surface antibody (anti-HBs) HBV core antibody (anti-HBc), and HBV-DNA quantitative levels. All patients were followed up at baseline, at the end of week 4 of anti-viral therapy, at the end of treatment and 12 weeks after treatment.

**Results:**

Assessment at 28 days showed significant decreases in ALT and AST levels in both groups, with stabilization of these levels on follow-up at 12 and 24 weeks. The efficacy of treatment was comparable in both groups. No case of ALT flare was observed in either group. Similar outcomes regarding AST and ALT levels were found in patients with diseases associated with immune derangement.

**Conclusion:**

The risk of resolved HBV reactivation during or after treatment with DAAs is low.

## Introduction

 Hepatitis B virus (HBV) and hepatitis C virus (HCV) co-infections are the leading causes of chronic liver disease and hepatocellular carcinoma worldwide. According to the World Health Organization, over 250 million people are currently infected with HBV, and more than 70 million with HCV. HBV and HCV co-infection is a complex clinical entity that has an estimated worldwide prevalence of 1–15%(1).

For the past two decades, the mainstay of antiviral therapy for CHC was a combination of pegylated interferon-α (peg-IFNα) plus ribavirin. This treatment was associated with low responses (overall 54-56% and significant toxicity that limited the widespread use of this therapy). The advances in antiviral drug discovery for CHC have led to the development of all oral IFN-free combinations of direct-acting antivirals (DAAs) that specifically target HCV proteins. These regimens have revolutionized HCV therapy, allowing extremely high cure rates in most individuals (>95%) with minimal adverse events (2).

In the 1970s, a new form of clinical HBV infection was reported in a patient with acute hepatitis, who was positive for anti-hepatitis B core (anti-HBc) immunoglobulin G (IgG), but negative for HBsAg (3). By developing highly sensitive molecular methods, the clinical entity of “occult” or “silent” HBV infection (OBI) was characterized (4). In an international workshop (2008) in Italy, researchers defined OBI as the detection of HBV DNA in the liver (with or without HBV DNA in serum) without HBsAg. OBI can be explained by the presence of HBV DNA in plasma or liver tissue with either seropositive or seronegative status. Seropositive OBI is characterized by the detection of the anti-HBc antibody with or without anti-HBs antibody, while undetectability of both anti-HBc and anti-HBs antibodies describe seronegative OBI (5). Resolved HBV infection was defined as the presence of a past HBV infection with positive HBc antibody, but undetectable serum HBV DNA and negative HBsAg.

Higher rates of OBI is reported among Egyptian chronic HCV, hemodialysis, children with malignancies, and cryptogenic liver disease patients. OBI prevalence in Egyptian HCV-positive patients is 1.85% to 38.3%, according to available data(6, 7).

HBV reactivation (HBVr) in patients with chronic hepatitis C during treatment with DAA drugs is possible because DAA drugs stop HCV replication and clear the virus from hepatocytes in weeks depending on the efficacy of the innate immune response. Hence the direct interference of HCV with the HBV replication is blocked suddenly, giving an intrahepatic replicative space for the HBV. Also, hepatocellular regeneration owing to HCV clearance may increase the pool of cells available for infection by HBV. This effect may have been less apparent with IFN based regimens due to the intrinsic anti-HBV activity of IFN(8).

The European Association for the Study of the Liver (EASL) recommends that HBV/HCV co-infected patients should be considered for treatment with nucleoside/nucleotide analogs for HBV when DAA treatment against HCV is indicated (9). Accumulating reports suggest that HBV reactivation following HCV eradication by interferon-free DAA treatment could occur in patients with isolated anti-HBc, not only in those with chronic hepatitis B and occult HBV infection (HBsAg negative, anti-HBc positive, HBV DNA detectable).

The risk of HBV reactivation during DAA treatment for HCV has been described by the American Association for the Study of Liver Diseases (AASLD)/ Infectious Diseases Society of America (IDSA) guidelines (10) and the Food and Drug Administration (11). So we aimed to assess and monitor the effect of DAA and HCV clearance on resolved HBV in a sector of our patients. 

## Methods

**Definitions**

**Reactivation of HBV (HBVr)**

HBV reactivation after DAA treatment was defined as a >1 log increase in serum HBV DNA accompanying by a ≥3-fold increase in baseline levels of alanine aminotransferase (ALT). Increases >1 log in HBV DNA levels that did not lead to clinical acute hepatitis (≥3-fold increase in ALT) were considered as HBV DNA flares of no clinical significance.

The clinical spectrum of hepatitis B flares varies from totally asymptomatic to symptomatic and to a feature similar to overt acute hepatitis (around 30%), with extreme manifestations of severe flares complicated by hepatic decompensation (jaundice and coagulopathy) or even leading to hepatic failure(12).

Resolved HBV infection was defined as the presence of a past HBV infection with positive HBc antibody, but undetectable serum HBV DNA and HBsAg with normal ALT levels.

The sustained virological response was defined as undetectable HCV quantitative titer after 12 weeks of completion of treatment.

**The study population**

**Figure 1:**
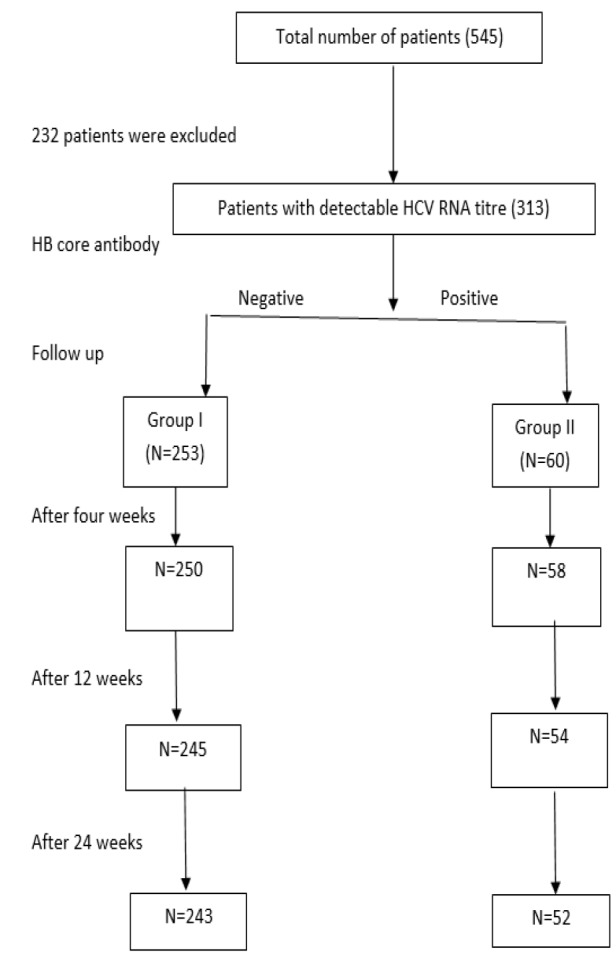
The study flow chart

The number of patients considered was 545 (figure 1). One patient with both HCV and overt HBV infection treated with dual direct antiviral drugs was excluded. Another patient with both HBV and HCV treated with interferon, and the direct-acting antiviral drug was excluded. The remaining patients that were not included in the present work were excluded due to a refusal to participate or presentation with an advanced stage of chronic liver disease or due to lack of patient’s data. Three hundred thirteen patients (all were genotype 4) were included in our observational and prospective study; 253 patient had chronic hepatitis C (CHC) only (group I), and 60 patients had both CHC and resolved HBV (group II). They all were eligible for treatment with DAAs therapy for chronic HCV in our hepatology unit, Internal Medicine Department, Zagazig University Hospitals from December 2017 to September 2018.

All patients received anti HCV treatment in the form of direct-acting antiviral therapy (Sofosbuvir (400mg)and Daclatasvir (60mg) ± ribavirin)according to the Egyptian guidelines for the treatment of HCV infection(13).

This study was done according to the institutional review board of Zagazig university hospitals.

**Exclusion criteria**

Patients with a history of HBV vaccination and overt HBV (HB surface antigen positive) or history of HBV treatment and any other contraindications for HCV treatment were excluded.

**All patients were subjected to**

1. Careful history taking
2. Thorough clinical examination
3. Pelvic-abdominal ultrasonography
4. Laboratory assessment in the form of:

- Complete blood count (CBC)
- Liver chemistry tests
- Kidney function tests
- PT, PTT, INR
- HCV antibody, HBsAg, HBs antibody, HCV RNA quantitative PCR using TaqMan technique, HBV core antibody total. HBV quantitative DNA PCR using quantitative real-time PCR was done for patients with HBV core positive test.
- The laboratory investigations were done before the beginning of treatment, and four weeks, eight weeks, 12 weeks during treatment, then these laboratory parameters were done after 12 weeks from completion of therapy.

**Statistical analysis**

All data were analyzed using MedCalc Statistical Software version 14.8.1 (MedCalc Software bvba, Ostend, Belgium; http://www.medcalc.org; 2014).

Continuous variables were expressed as the mean ± SD, median and range according to the normality of the data where customarily distributed data were expressed as mean ± SD, whereas non-normally distributed data were expressed as median and (min-max). The categorical variables were expressed as a number (percentage). Continuous variables were checked for normality by using the Kolmogorov Smirnov test.

All normally distributed data were analyzed using Independent Student t (t) test between two independent groups. Data found to be non-normally distributed were analyzed using the Mann-Whitney U (MW) test.

Categorical variables were compared using the Chi-square (χ2) test. For paired data, customarily distributed ones were analyzed using Paired t (t) test. Data found to be non-normally distributed were analyzed using the Wilcoxon signed-rank test (WT). P <0.05 was considered statistically significant (S), and P ≥ 0.05 was considered none statistically Significant (NS). 

## Results

Our study included 313 patients with chronic hepatitis C (CHC) infection aged 53.28±11.89 years. We classified patients into two groups: group I (n=253) included CHC patients only while group II included CHC and isolated anti-HBc +positive patients (n=60). Characteristics of patients including demographic and laboratory data where there was a statistically significant difference between the two groups as regards hemoglobin level, total bilirubin, AST and serum creatinine (p<0.02, 0.04, 0.02 and 0.049 respectively), quantitative HBV DNA PCR was undetectable in group II (table 1). After four weeks from the start of treatment with DAAs, the follow-up laboratory workup was done for both groups (group I (n=250) and group II (n=58)). At every stage of follow up, patients who missed follow up or patients with incomplete laboratory data were excluded. In group I there was a highly significant difference in hemoglobin level, ALT and AST before and after 28 days from the start of treatment with DAAs (P< 0.005, 0.0001, 0.0001 respectively). On the other hand, in group II there was a statistically significant difference in direct bilirubin level, ALT and AST before and after 28 days of treatment (P< 0.02, 0.001, 0.002 respectively). HCV quantitative PCR was undetectable in all patients in both groups (<15 (IU/mL) except in three patients in group I where there is a marked reduction of HCV PCR titer (partial remission). HBV DNA PCR for group II was undetectable after four weeks except in one patient where there was a detectable number of 264IU/ml but without an increase in the liver enzymes or bilirubin level. Also, the patient clinically was free, and no treatment for HBV detectable level was given (table 2). 

At the end of treatment (EOT), follow up laboratory investigations in group I (n=245) in comparison with previous laboratory parameters at four weeks revealed a statistically significant difference as regards ALT (P=0.05). On the other hand, in group II (n=54), there was a statistically significant difference as regards the direct bilirubin level before and after the EOT (P=0.05). All patients in both groups had undetectable HCV quantitative PCR titer. All patients in group II had undetectable HBV DNA PCR level / (<12IU/ ml) (table 3). As regards the laboratory changes after 24 weeks (SVR12) in comparison with the same laboratory data at the EOT in group I (n=243), there was a statistically significant difference of the serum level of total bilirubin (p=0.01) all patients in group II had undetectable HBV DNA PCR level (<12iu ml), HCV quantitative titer was undetectable in both groups. Also, HBV quantitative DNA titer was undetectable in group II (table 4). Two hundred twenty-nine patients had no history or evidence of chronic illness, while only 24 patients had a history of chronic diseases, such as diabetes mellitus, thyroid disorders, vitiligo, and steroid therapy. Comparison between the two groups showed no statistically significant difference as regards all baseline laboratory data (table 5). Concerning the group with chronic illness (n=24), there was an improvement in the liver enzymes (ALT, AST) after treatment but this improvement was not statistically significant (P=0.16, 0.6 respectively) (table 6).

**Table 1:** Comparison between baseline demographic and laboratory characteristics of the studied groups

		Group I (N=253)	Group II (N=60)	P
Age (Years)		55 (8 – 75)	60 (20 – 69)	0.002
Sex	Male	155	24	0.02
	Female	95	30	
Other conditions	Diabetes	17	8	
	Thyroid disorders	2	0	
	Vitiligo	2	0	
	Steroid therapy	1	0	
HCV RNA (IU/mL)		472000 (36.2 – 14932074)	538230 (120 – 6692647)	0.75
WBCs (x 10^9^)		6.1 (1.9 – 21)	6.15 (1.5 – 14.5)	0.91
Hemoglobin (g/dL)		12.57 ± 2.07	11.75 ± 2.17	0.02
Platelets (x 10^9^)		150 (13.5 – 442)	157.5 (33 – 346)	0.68
Total Bilirubin (mg/dL)		0.84 (0.09 – 17.1)	0.92 (0.3 – 4.4)	0.04
Direct Bilirubin (mg/dL)		0.21 (0.02 – 14.8)	0.22 (0.1 – 2)	0.22
Albumin (g/dL)		3.9 (2 – 5.04)	3.7 (2.3 – 4.4)	0.07
ALT (mg/dL)		39 (6.2 – 370)	33.96 (11 – 386)	0.26
AST (mg/dL)		41 (10 – 263)	30 (10.5 – 174)	0.02
INR		1.19 ± 0.18	1.24 ± 0.21	0.14
Creatinine (mg/dL)		0.89 (0.4 – 2.1)	0.91 (0.67 – 1.7)	0.049

**Table 2:** Laboratory changes in both groups before and after 28 days

	Group I			Group II		
	Before(n=250)	After(n=250)	P	Before(N=58)	After(N=58)	p
WBCs (x 10^9^)	5.7 (2 – 16)	5.5 (1.2 – 17.7)	0.61	6.4 (1.5 – 12.7)	5.6 (2.05 – 17.8)	0.57
Hemoglobin (g/dL)	12.43 ± 2.12	12.06 ± 1.94	0.005	11.55 ± 1.88	11.31 ± 1.91	0.51
Platelets (x 10^9^)	152 (29 – 442)	154 (12.4 – 371)	0.81	159 (46 – 346)	166 (41 – 270)	0.52
Total Bilirubin (mg/dL)	0.9 (0.3 – 8.99)	0.86 (0.3 – 9.16)	0.34	0.9 (0.3 –4.4)	0.8 (0.4 – 2.7)	0.08
Direct Bilirubin (mg/dL)	0.22 (0.07 –2.3)	0.23 (0.1 – 2.5)	0.38	0.28 (0.1 – 2)	0.17 (0.07 – 1.6)	0.01
Albumin (g/dL)	3.8 (2.2 – 5)	3.84 (2.4 – 5.3)	0.86	3.8 (2.3 – 4.4)	3.75 (3.2 – 4.3)	0.34
ALT (mg/dL)	36 (6.2 – 200)	27 (8 – 100)	<0.0001	38 (17 – 72)	17 (15 – 36.6)	0.001
AST (mg/dL)	40 (10 – 263)	30 (3 – 116)	<0.0001	33 (18 – 88)	23 (11 – 43)	0.002
INR	1.21 ± 0.17	1.18 ± 0.18	0.26	1.27 ± 0.25	1.20 ± 0.21	0.16

**Table 3:** Laboratory changes in both groups before and after 12 weeks

	Group I		P	Group II		p
	At 28 days (N=245)	After 12 weeks (N=245)		At 28 days (n=54)	After 12 Weeks(n=54)	
WBCs (x 10^9^)	5.4 (2 – 17.7)	5.05 (2.4 – 14.6)	0.80	5.6 (2.1 – 11)	5.5 (2.3 – 8)	0.08
Hemoglobin (g/dL)	11.62 ± 2.38	11.82 ± 1.57	0.38	11.15 ± 2.16	10.84 ± 2.37	0.37
Platelets (x 10^9^)	151 (44 – 300)	143 (44 – 362)	0.25	143 (41 – 270)	141 (25 – 241)	0.62
Total Bilirubin (mg/dL)	0.8 (0.3 – 9.2)	0.8 (0.4 – 3.5)	0.60	0.8 (0.4 – 2.74)	0.85 (0.3 – 3.96)	0.43
Direct Bilirubin (mg/dL)	0.21 (0.1 – 2.15)	0.24 (0.1 – 3.04)	0.87	0.19 (0.07 – 1.6)	0.26 (0.1 – 2.4)	0.02
Albumin (g/dL)	3.89 (2.4 – 5.3)	3.6 (0.82 – 4.7)	0.65	3.48 (2.65 – 4.1)	3.64 (2.54 – 4.2)	0.64
ALT (mg/dL)	26 (0.9 – 100)	20 (10 – 60)	0.009	17.5 (15 – 70)	20 (12 – 41)	0.80
AST (mg/dL)	30 (3 – 116)	26 (2.1 – 70)	0.09	22.9 (11 – 75)	26.1 (10 – 78)	0.46
INR	1.19 ± 0.15	1.19 ± 0.17	0.91	1.24 ± 0.20	1.23 ± 0.18	0.91

**Table 4:** Laboratory changes in both groups before and after 24 weeks

	Group I			Group II		
	At 12 weeks (n=243)	After 24 weeks (n=243)	P	At 12 weeks(N=52)	After 24 weeks(N=52)	p
WBCs (x 10^9^)	4.5 (3.2 – 12.6)	4.1 (2.9 – 11.7)	0.92	5.5 (1.9 – 6.6)	6.2 (2.2 – 9.2)	0.15
Hemoglobin (g/dL)	12.03 ± 1.51	11.05 ± 1.97	0.1	10.85 ± 2.13	10.45 ± 2.31	0.26
Platelets (x 10^9^)	130 (56 – 362)	102 (13 – 360)	0.52	142 (38 – 241)	96 (61 – 187)	0.17
Total Bilirubin (mg/dL)	0.91 (0.4 – 3.5)	1.26 (0.58 – 4.3)	0.01	0.79 (0.45 – 3.9)	1.1 (0.3 – 2.4)	0.32
Direct Bilirubin (mg/dL)	0.4 (0.1 – 1.3)	0.77 (0.2 – 1.3)	0.11	0.3 (0.1 – 2.4)	0.3 (0.1 – 1.8)	0.46
Albumin (g/dL)	3.5 (0.8 – 4.6)	3.49 (2.5 – 4.8)	0.62	3.6 (2.5 – 4.2)	3.6 (2.1 – 4.8)	0.99
ALT (mg/dL)	17 (10 – 60)	21 (11– 52)	0.36	24 (12 – 41)	27 (10 – 33)	0.73
AST (mg/dL)	21 (17 – 70)	34 (15 – 65)	0.50	20 (10 – 78)	31 (20 – 42)	0.43
INR	1.22 ± 0.22	1.05 ± 0.47	0.23	1.34 ± 0.15	1.35 ± 0.26	0.83

**Table 5:** Comparison of laboratory data in patients with and without chronic illness

	No chronic illness (N=229)	With chronic illness (N=24)	P
WBCs (x 10^9^)	6 (1.5 – 21)	6.88 (3.6 – 14.5)	0.07
Hemoglobin (g/dL)	12.47 ± 2.06	12.10 ± 2.48	0.41
Platelets (x 10^9^)	151 (13.5 – 442)	167 (49 – 301)	0.73
Total Bilirubin (mg/dL)	0.85 (0.09 – 8.99)	1 (0.37 – 17.1)	0.11
Albumin (g/dL)	3.82 (2 – 5.04)	3.75 (2.3 – 4.6)	0.37
ALT (mg/dL)	37 (6.2 – 386)	43 (16 – 122)	0.15
AST (mg/dL)	39 (10 – 263)	49 (17 – 193)	0.14
INR	1.20 ± 0.18	1.18 ± 0.22	0.66
Creatinine (mg/dL)	0.89 (0.4 – 2.1)	0.94 (0.7 – 1.5)	0.07

**Table 6:** ALT and AST changes patients with chronic illnesses before and after treatment (SVR12)

	Before (N=24)	After (N=24)	P
ALT (mg/dL) Median (Range)	37.5 (29 – 99)	32 (10 – 36)	0.16
AST (mg/dL) Median (Range)	58.5 (30 – 88)	39.5 (12 – 46)	0.06

## Discussion

Viral hepatitis is one of the most common infectious diseases worldwide. According to WHO, viral hepatitis caused 1.34 million deaths in 2015, a number comparable to the deaths caused by tuberculosis and higher than those produced by HIV(14). The prevalence of hepatitis C virus (HCV) infection in Egypt is the highest in the world (15). The introduction of direct-acting antiviral (DAA) treatment for HCV has led to a transformation in the management of HCV worldwide. HBV infection is still another healthcare problem. Combined HCV and HBV infection represent a challenge in clinical practice. The EASL and AASLD have issued guidelines for the management of HCV and HBV confection (16, 17).

Treatment with DAA for HCV in patients who are HBV Ag positive has led in various studies to HBV reactivation with increased DNA replication and increased ALT level. However, the clinical impact was not marked except in cases where accompanying immunosuppression was present. HBV therapy with nucleotide(s) analogs was introduced upon reactivation, which prevented the recognition of the natural history of this reactivation in the absence of intervention (18). HBV reactivation with ALT flare is usually feared due to the possibility of precipitating severe or fulminant hepatitis (19). The FDA has introduced a requirement on DAA for a label concerning the risk of HBV reactivation (20).

HCV and HBV co-infection usually leads to dominant suppression of HBV replication and reduced expression of HBV markers. Our study is conducted to clarify the effect of HCV treatment with DAA on resolved HBV infection in a clinical practice environment.

The current study is carried out on two groups of HCV patients. Group I consists of HCV patients without HBV infection. Group II includes HCV patients with resolved HBV co-infection. All patients were assessed at baseline for personal demographic, clinical, biochemical, and molecular characteristics. DAA therapy was initiated in all cases. Reassessment for all patients was done after 28 days, 12 weeks, and 24 weeks for biochemical measures of liver inflammation and function. Also, HCV quantitative PCR and HBV quantitative PCR were checked at the same intervals. Both groups were comparable at baseline in most characteristics with slightly small differences in average age, sex, serum hemoglobin, AST, and creatinine levels. Diabetes mellitus was common in group II. Whereas thyroid disease and vitiligo were present in some group I patients. No significant difference was present at baseline regarding HCV-RNA levels.

As expected, assessment at 28 days showed significant decreases in ALT and AST levels in both groups, with stabilization of these levels on follow-up at 12 and 24 weeks. The efficacy of treatment was comparable in both groups. No case of ALT flare was observed in either group. Similar outcomes regarding AST and ALT levels were found in patients with diseases associated with immune disorder. This was aligned with other studies which showed that HBV reactivation in cases of resolved HBV receiving DAA for HCV was very mild, manifesting only by increased DNA replication detectable only by very sensitive assays. No clinical impact was detected in such cases in line with findings by other studies (21).

The mechanism of HBV reactivation with HCV treatment is not settled. HCV clearance is associated with a positive impact on the immune system with improvement in immunologic disorders(21). The occurrence of HBV reactivation suggests the contribution of different mechanisms, such as an increase in a previously limited replicative space, or the appearance of an immune reconstitution targeting HBV. More research is expected to clarify these pathophysiologic mechanisms. 

One of the strengths of this study is that it is a prospective work, with a relatively large number of HCV and resolved B virus in comparison to previous studies. Also, monitoring of patients for a long time after the end of treatment with measurement of HBV DNA level.

The current study shows that monitoring with biochemical measures of inflammation will be appropriate to detect any clinically significant reactivation, which is to be supplemented by additional markers. The probability of occurrence of HBV reactivation in resolved HBV appears to be low. Assessment of HBV status before starting DAA therapy is as always generally warranted. In conclusion, HBV assessment should be part of the routine evaluation before DAA therapy, with attention to any enzymatic flare in the presence of HBV co-infection taking into consideration that this possibility is not likely with resolved HBV infection.

## Conflict of interests

The authors declare that they have no conflict of interest.
